# A new technical solution to the problem of increasing the resolution of X-ray diffraction methods

**DOI:** 10.1107/S1600576724011130

**Published:** 2025-02-01

**Authors:** H. R. Drmeyan, S. A. Mkhitaryan, A. H. Mkrtchyan

**Affiliations:** a Institute of Applied Problems of Physics, National Academy of Science of the Republic of Armenia, 25 Hrachya Nersisyan Str., Yerevan, Armenia, 0014; SLAC National Accelerator Laboratory, Menlo Park, USA

**Keywords:** X-ray interferometers, X-ray diffraction patterns, resolution, scanning, single crystals, X-ray pattern enlargement

## Abstract

A new technical solution to the problem of increasing the resolution of X-ray diffraction methods has been proposed and implemented, based on linearly enlarging the X-ray topographic patterns. For implementation of the suggested method, a novel device has been developed, created and tested that makes it possible to scan synchronously the slit for transmitting separate parts of the X-ray diffraction pattern and the X-ray film with a predetermined speed ratio. The possibility of significantly increasing the resolution of X-ray diffraction patterns with the suggested new scanning method has been experimentally proven. It was shown that if individual parts of the diffracted beam are passed successively through a narrow slit, which is synchronously scanned along with the X-ray detecting film, we obtain an enlargement in topographic patterns. A proposed scheme for enlarging the image in parts and a description and the operating principle of the scanning device are also presented. The relationship between the ratio of the speeds of the slit and the X-ray film movement and the parameters of the scanning device and the sample (slit width, total thickness of thin crystals, thickness of a thick crystal *etc*.) was revealed. The speeds of the reciprocating motion of the slit and the X-ray film were calculated. It has been experimentally proven that the scanning process does not introduce new information into the interference pattern but only enlarges it, since these patterns in sectional topograms differ only in size in the scattering plane.

## Abstract 2

A new method of diffraction microradiography of single crystals is proposed with the aim of increasing the resolution of X-ray topographic patterns. For the implementation of this method, a device for synchronous scanning of the slit for transmitting separate parts of the X-ray diffraction pattern and the X-ray film with a predetermined speed ratio is designed, created and tested.

## 1. Introduction

X-ray diffraction has been used for many years to study imperfections in the structure of crystals. For this purpose, several methods have been developed, based on the fact that small angular deviations or differences in the lattice parameters of crystalline plates are revealed as a fine structure of diffraction spots. We will not consider all these methods, since they do not provide a direct image of the dislocation structure. We will highlight only those methods that make it possible to obtain images of individual dislocations.

To date, various ‘direct’ observation methods of dislocations have been developed and successfully applied. The following can be noted from the most common methods:

(1) Methods based on the use of a reflected beam, for example, the Newkirk method (Newkirk, 1959 ▸), which is a modified Berg–Barrett method (Newkirk, 1958 ▸).

(2) Methods based on the use of a transmitted beam, where most of the structural details are obtained using the technique proposed by Lang (1959 ▸).

(3) Double-crystal goniometer methods, where a particularly accurate and sensitive method was developed by Bonse & Kappler (1958 ▸).

(4) An anomalous transmission method developed by Bormann *et al.* (1958 ▸). Here the crystal is chosen to be thick enough that the normal absorption is high. If the beam falls at the exact angle of Bragg reflection, then the energy flow is directed along the atomic planes, and the anomalous passage of standing waves is observed along these planes.

(5) A combination of Bormann and Lang methods, which was used by Barth & Hosemann (1958 ▸) and Authier (1960 ▸).

There are also several methods based on X-ray diffraction in different crystalline systems, for example in double-crystal and triple-crystal interferometers (Authier *et al.*, 1968 ▸; Bezirganyan & Drmeyan, 1985 ▸; Chen *et al.*, 2003 ▸; Lider, 2021 ▸; Drmeyan, 2022 ▸). Particularly, the review paper by Lider (2021 ▸) describes different X-ray diffraction topography methods for studying various structural defects in crystalline materials. However, X-ray diffraction methods, widely used in identifying structural imperfections in crystalline materials, sometimes have limited capabilities due to their insufficient resolution.

Resolution is determined, on the one hand, by the width of the dislocation image and, on the other hand, by the resolution of the photographic emulsion. Since the X-ray methods discussed above in practice do not provide image enlargement (zooming), on the photographic plate the dislocation distribution in the sample is recorded at its natural size, which requires high resolution of the emulsion.

The question of enlarging the image of structural imperfections in crystalline materials, at first glance, seems impossible in connection with the fact that the refractive index of X-rays differs slightly from unity (X-rays are refracted slightly). Because of this, methods for enlarging X-ray diffraction patterns were not developed until recently, and X-ray optical magnifiers (lenses) did not exist.

Previously (Mkhitaryan *et al.*, 2024 ▸), we proved theoretically and experimentally that X-ray topographic patterns can be enlarged by passing an X-ray beam containing information about micro defects in the structure (dislocations) of the single crystal under study through an ideal thick (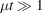, where 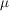 is the linear absorption coefficient and *t* is the crystal thickness) crystal located in the reflection position. However, the magnification in such systems does not exceed two orders. The enlargement achievable using this method is actually determined by the ratio of the thicknesses of the thick perfect single crystal and the sample under study. Substantial enlargement cannot be achieved in this way due to the inevitable absorption of X-rays in a thick crystal. Consequently, the study of the problem of increasing the resolution of X-ray diffraction methods is relevant for condensed matter physics and materials science.

We propose a new method for enlarging X-ray topographic patterns, which can be achieved by passing separate parts of a diffracted beam successively through a narrow slit, that is, by increasing the magnification of the image in parts. In this work, we also present a scheme for enlarging the image in parts, as well as a description and the principle of operation of the scanning device.

## 2. Theoretical reasoning

X-ray topographic methods for direct observation of defects in crystals use dynamic X-ray diffraction. It is known (Authier, 2001 ▸) that during X-ray diffraction in crystals, a strong angular expansion of the beam occurs. This angular enlargement of beams can be used to produce linear enlargement of X-ray patterns, which makes it possible to increase the resolution of X-ray diffraction patterns.

An analytical calculation of wave propagation in crystals during dynamic X-ray diffraction is given by Authier (2001 ▸). It is shown that during X-ray diffraction in crystals a strong angular increase in the beam is observed. This increase is expressed by the formula
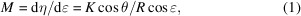
where dɛ is the angle of convergence of the incident beam in the crystal, dη is the angle of divergence of the beam, θ is the Bragg angle, *R* is the radius of the dispersion surface and *K* is the wavenumber (*K* = 1/λ).

**Figure 1:**
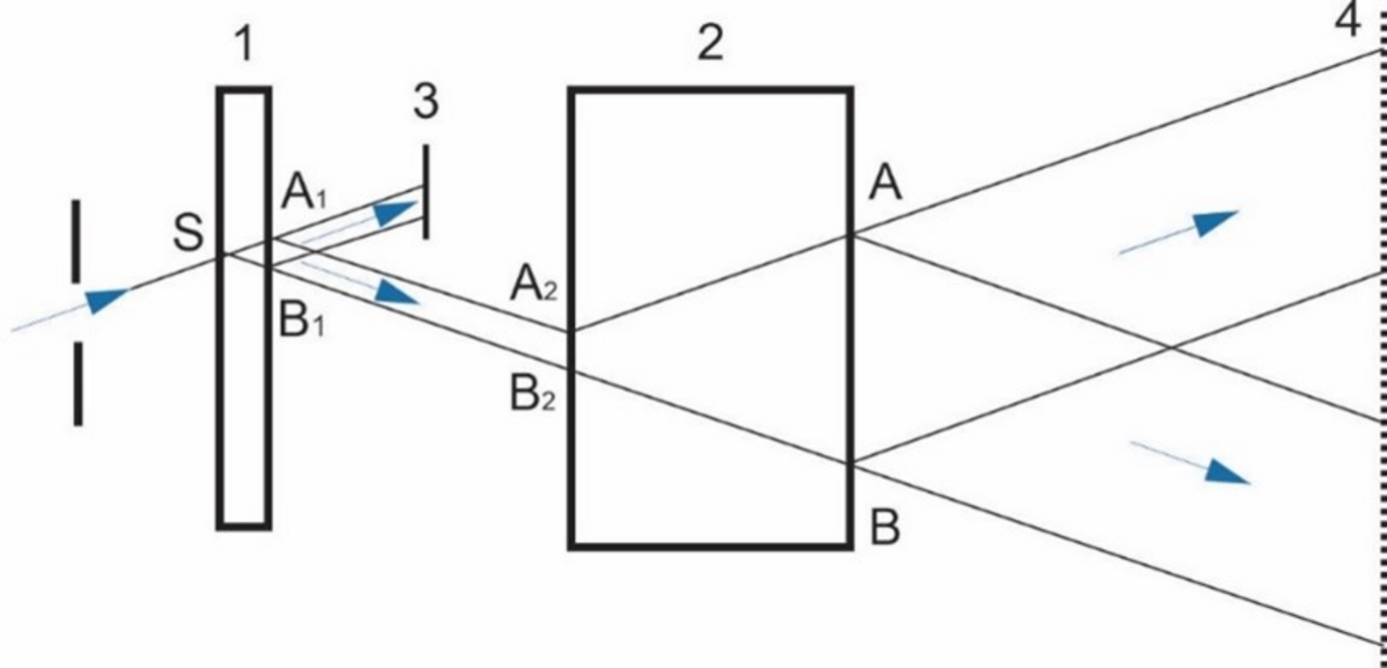
Scheme for enlarging X-ray diffraction patterns, where 1 is the crystal under study, 2 is a thick perfect crystal, 3 is a screen to delay the transmitted beam and 4 is an X-ray plate.

It follows from (1) that the crystal is a powerful magnifier, and for 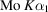 radiation and silicon reflection 2
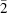
0, *M* has a value of about 10^3^. Indeed, if a beam containing an X-ray topographic pattern obtained from a thin sample under study is passed through a thick perfect crystal located in the reflection position, then a linear enlargement in the topographic pattern occurs (Fig. 1 ▸).

As can be seen from Fig. 1 ▸, the linear enlargement is small: the interference pattern is limited within the triangle 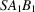 and the width of the image is no more than the base 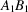 of this triangle. The increase is determined by the ratio 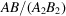 or 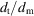, where 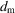 is the total thickness of thin crystals and 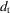 is the thickness of the thick crystal, and it can be increased by decreasing 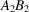. The latter can be achieved by successively passing separate parts of the diffracted beam through a narrow slit, *i.e.* enlarging the image part by part.

**Figure 2:**
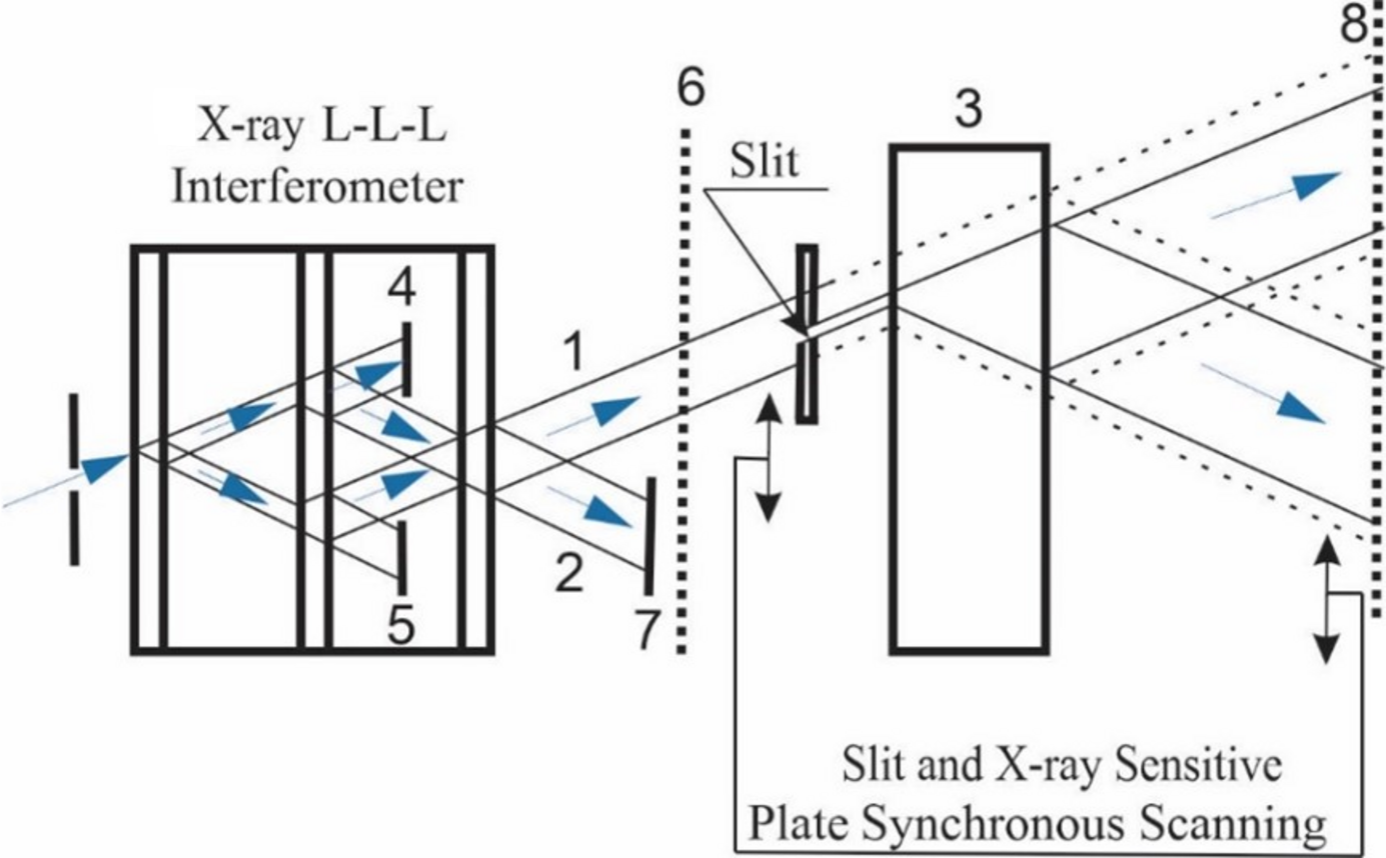
A scheme for a setup that allows us to enlarge the X-ray diffraction pattern in parts, where 1 and 2 are transmitted and diffracted beams at the output of the three-crystal system, 3 is a thick perfect single crystal located in the reflection position, 4, 5 and 7 are delay screens, and 6 and 8 are X-ray plates.

If the diffraction pattern is enlarged in parts—that is, its separate parts are passed through a narrow slit, as shown in Fig. 2 ▸—then we obtain additional enlargement. The system of thin single crystals shown in Fig. 2 ▸ is a three-block triple Laue (L-L-L) interferometer, with which we obtain a moiré pattern. A narrow beam of X-rays, passing through a collimator with a diaphragm, falls on the interferometer. Beams 1 and 2 at the output of the three-crystal system, which contribute to the moiré pattern, fall on a thick perfect single crystal 3, which is in the reflection position, and beams that do not participate in the formation of the moiré pattern are delayed by screens 4 and 5. During the experiments, X-ray patterns were obtained on X-ray plates 6 and 8. For clarity, Fig. 2 ▸ shows only the path of rays in a thick crystal for beam 1 (beam 2 is delayed by screen 7). The dashed lines after the slit show the path of rays in a thick perfect crystal in the absence of a slit.

A diffracted X-ray beam that contributes to the moiré pattern is passed in parts sequentially along the width of a narrow scanning slit installed between a system of thin single crystals and a thick single crystal, and is recorded on the X-ray film, which is located behind the thick perfect single crystal. The X-ray film is scanned synchronously with the slit by a speed ratio consistent with the system parameters 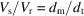, where 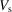 and 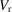 are the speeds of movement of the slit and the X-ray film, respectively.

Thus, when the slit is scanned, it gradually transmits the individual parts of the beam diffracted in the first crystal (or in a system of thin crystals), which, passing through a thick perfect single crystal in the direction of reflection, undergo an angular enlargement.

If the scanning speeds are chosen so that their ratio satisfies the condition
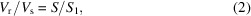
where *S* is the base of the Bormann triangle (segment *A*_1_*B*_1_ in Fig. 1 ▸) and 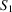 is the width of the slit, *i.e.* the width of the beam incident on a thick ideal single crystal, then on the X-ray film we will obtain an enlarged image of the topogram or various patterns of dynamic effects.

The proposed method and device make it possible to synchronously scan the slit and the film with predetermined (calculated) speed ratios and, as a result, have enlarged (expanded) images of individual parts of the *A*_2_*B*_2_ beam on the X-ray film that are linked to each other.

Such a system provides a significant magnification in the diffraction pattern, *i.e.* it allows us to significantly increase the resolution of X-ray diffraction methods. The proposed method makes it possible to increase the resolution by at least another additional order, *i.e.* the suggested new method makes it possible to ultimately enlarge X-ray diffraction patterns by three orders.

## 3. Description of the scanning device and experiment

**Figure 3:**
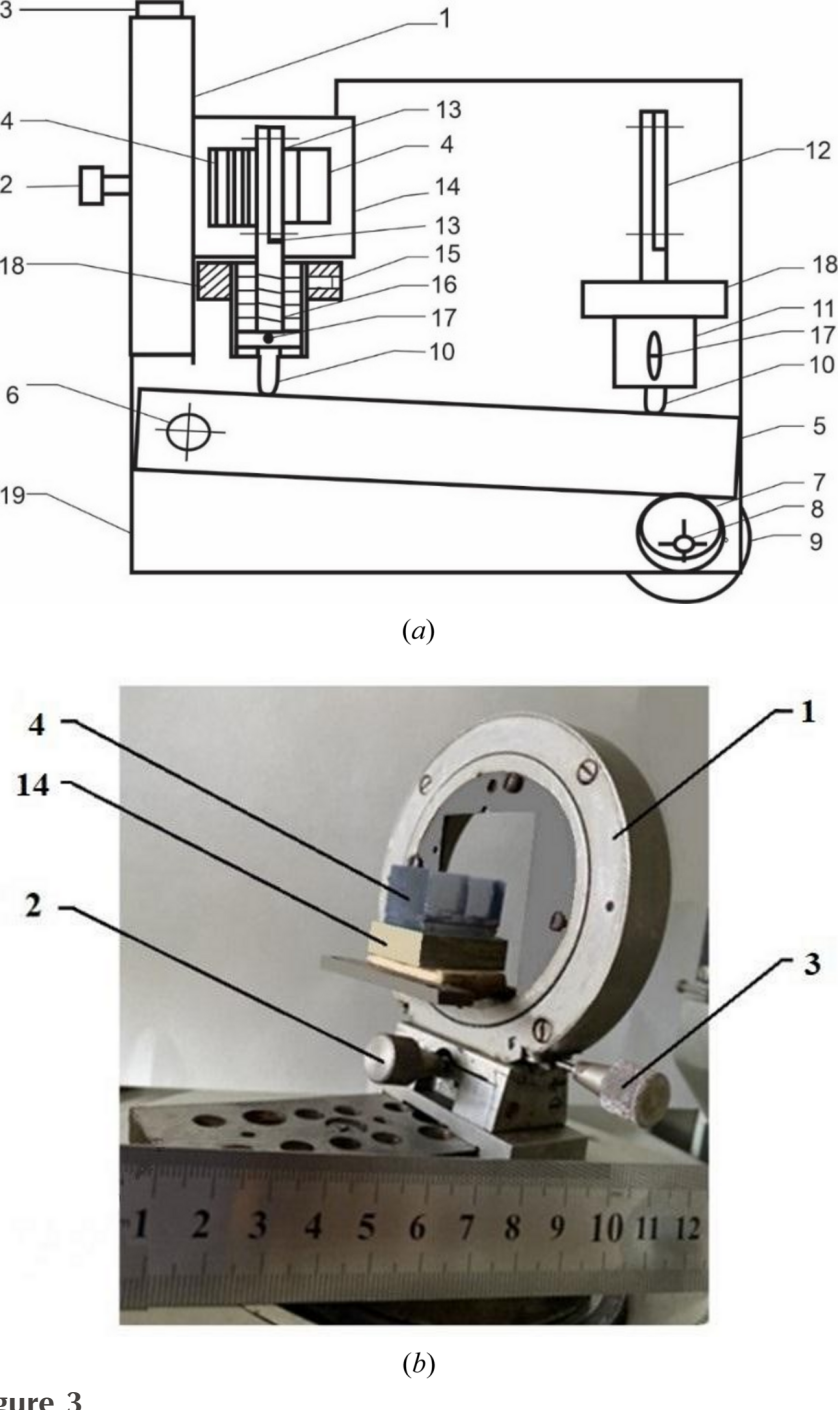
(*a*) Schematic representation of the scanning device. (*b*) Axonometric picture of the goniometric head with monolithic three-block L-L-L X-ray interferometer: goniometric head (1), aligning micro screws (2) and (3), X-ray monolithic three-block interferometer (4), and table (14).

The scanning device for obtaining high-resolution X-ray diffraction patterns is presented in Fig. 3 ▸(*a*). It consists of a goniometric head 1 [an axonometric picture is separately presented in Fig. 3 ▸(*b*)] which can be adjusted in two mutually perpendicular directions using micro screws 2 and 3. A table 14 is attached to the head 1, on which the test sample 4 is placed.

In Fig. 3 ▸, the studied sample of the diffraction system is a three-block L-L-L interferometer consisting of three thin blocks equidistant from each other and an additional fourth thick block (enlarger). Lever 5, which can freely rotate around the vertical axis 6, is set in motion (rotation around an axis in the horizontal plane) using an eccentric (cam) 7 mounted on a vertical axis 8, to the other end of which a pulley 9 is mounted. To rotate pulley 9, we use the electric motor of the scanning device of the Lang Raman diffraction chamber for X-ray structural analysis, on which the described device is placed during the entire X-ray experiment. Using a belt drive, the rotation of the pulley mounted on the axis of the electric motor is transmitted to the pulley 9. During the rotation, pulley 9 rotates eccentric 7, which in its turn creates uniform movement of lever 5. Piston pushers 10, rigidly connected to pistons 11, touch the lever. At the other ends of the pistons, devices for placing a cassette with photographic film 12 and a screen with a narrow vertical slot 13 are rigidly attached. The piston–pusher systems are brought to the zero-position relative to the lever using screws 15, corresponding to the case when the pushers are in contact with the lever. The location of these systems is selected from condition (2), and the speeds 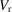 and 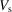 are easily calculated using the distances of the points of contact of the piston pushers 10 on lever 5, calculated from the axis of rotation of the lever 6. During the reciprocating movement of the lever (the lever approaches the same eccentric focus, around which the eccentric itself rotates), for the piston to return back and remain pressed against the lever all the time, springs 16 are placed above the pistons. The pistons move inside the cylinder with guide pins 17, which with holders 18 are rigidly attached to the base 19. The entire system is designed so that it can be adapted to a Lang Raman type diffraction chamber, with which the sample is adjusted to a precise Bragg angle relative to the incident primary beam.

All experiments were performed on a Lang A-3 type chamber (produced by Japanese company Rigaku Denky) with usage of unpolarized 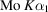 radiation. During the experiments, X-ray quanta with energies from 6.5 to 11.5 keV were used.

The alignments of thin and thick crystals are carried out during the sample preparation process. The equality of the distance between the blocks was measured by optical instruments with an accuracy of 1 µm, and the alignment was achieved by grinding the sample blocks with fine diamond powder with a grain diameter of 3 µm. After subsequent mechanical polishing, the manufacturing process ends with chemical etching/chemical polishing, after which we obtained a system with very precisely manufactured and aligned crystals. Chemical etching completely removes the damaged upper layer of the crystal blocks, and therefore the stress in the crystal blocks that arises during grinding. In general, the requirements for the manufacturing accuracy of the studied system were taken into account and were quite acceptable in the context of their practical implementation and creation of the studied samples. This issue is discussed in more detail in the article by Mkhitaryan *et al.* (2024 ▸). In addition, the manufactured crystal system (sample) was monolithic, and all crystalline blocks were rigidly connected to a common thick base and were in a vertical position; hence the occurrence of stress in the crystals was excluded. During the experiments, the obtained ideal geometry of the sample was not disturbed.

**Figure 4:**
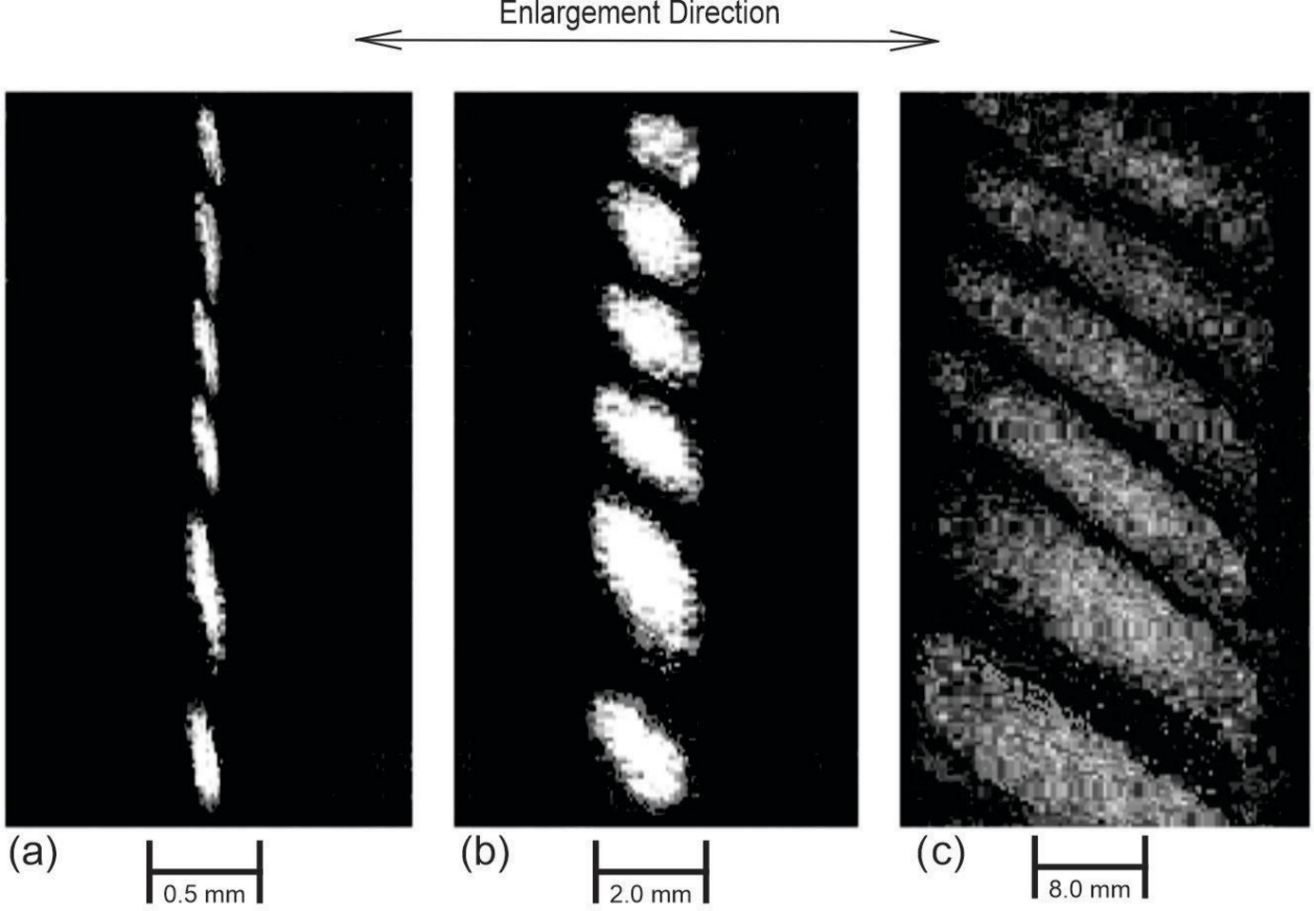
Moiré patterns: (*a*) formed in an X-ray three-crystal system; (*b*) enlarged without scanning the slit and X-ray film; (*c*) enlarged during the synchronous scanning of the slit and X-ray film.

Fig. 4 ▸(*a*) shows the moiré pattern formed by a three-crystal system (Fig. 2 ▸). The image was obtained on X-ray film 6, placed between the third and fourth blocks. Fig. 4 ▸(*b*) shows the same pattern recorded on X-ray film 8 after the enlargement (after passing through the fourth block), without scanning the slit and with a stationary X-ray film (Fig. 2 ▸).

Fig. 4 ▸(*c*) shows the same pattern recorded on X-ray film 8 when it is scanned synchronously with the slit according to the speed ratio specified in (2). As can be seen from Fig. 4 ▸(*b*), when beams contributing to moiré patterns pass through a thick crystal located in the reflection position, these patterns are zoomed in. As can be seen from Fig. 4 ▸(*c*), the enlargement effect is much stronger in the presence of a scanning slit.

Thus, the scheme shown in Fig. 2 ▸ actually represents an X-ray magnifier. Similarly, it is possible to obtain an enlargement in interference patterns obtained from different types of interferometers and also to increase the resolution of X-ray topographic methods.

This effect can be used in such areas of physical research as X-ray diffraction of micro defects, X-ray spectroscopy, X-ray interferometry and precision X-ray diffraction analysis, as well as for studying the fine structure of interference patterns.

The optimal exposure time in our experiments was selected to be approximately 12–14 h. When this time was exceeded to increase the contrast of the pattern in Fig. 4(*c*) obtained from position 8 with scanning (Fig. 2 ▸), the contrast of the pattern obtained from position 8 without scanning worsened in the case of simultaneous recording at two films in positions 6 and 8 (Fig. 2 ▸). In addition, an excessive increase in the exposure time could lead to distortions of the interference patterns at all positions due to possible additional temperature gradients, sample drifts relative to the incident beam and other side effects. With the same exposure time, the pattern contrast at position 8 [Fig. 2 ▸ and Fig. 4 ▸(*c*)] turned out to be lower than that at position 6. The contrast of the pattern in Fig. 4 ▸(*c*) could be improved by scanning the slit and the film more slowly, but the scanning device we propose was driven by the electric motor of the scanning device of the Lang A-3 chamber, which has a standard scanning speed of 20 mm h^−1^.

## 4. Results, discussion and conclusion

It may seem that the interference patterns observed after the enlarger (an ideal thick crystal) did not exist before it and were formed in it, that is, the last crystal does not play the role of an enlarger but participates in the process of formation of these patterns.

The fact that the last thick crystal (magnifier) only increases the linear dimensions of the diffraction pattern and does not introduce any additional information into the interference pattern can be seen from the following theoretical considerations (reasonings) and experimental facts:

(1) The enlarger crystal is thick and ideal and has a large absorption coefficient. Therefore, the wavefield completely disappears, and no *Pendellösung* occurs in it. Consequently, the distribution of the field inside the crystal, which has a period equal to the interplanar distance of the reflecting planes, is not preserved outside the crystal (von Laue, 1960 ▸).

(2) Furthermore, since the magnifying crystal is perfect, and no images of defects are observed in its topogram, it follows that the magnifying crystal does not change the nature of the intensity distribution in the beam passing through it. As a result, it reduces the overall intensity of a diffracted beam without changing the interference pattern, but increases its linear dimensions in the scattering plane. Experiments have also shown that the interference pattern is not created by the thick block crystal, since if one of the thin blocks of the interferometer is removed, the interference pattern observed after the thick block crystal disappears.

(3) Comparing the sectional moiré patterns shown in Figs. 4 ▸(*a*) and 4 ▸(*b*), it is easy to see that they differ in size only in the scattering plane, *i.e.* the thick crystal only plays the role of a linear enlarger.

Comparing the sectional moiré patterns shown in Figs. 4 ▸(*b*) and 4 ▸(*c*), obtained without and with synchronous scanning of the slit and X-ray film, we can state that the scanning process does not introduce new information into the interference pattern, but only enlarges its linear dimensions in the scattering plane.

The proposed device makes it possible to synchronously scan the slit and X-ray film with the predetermined speed ratio.

Experimental results show that, with the help of the proposed scanning method, it is possible to increase the linear dimensions of X-ray diffraction patterns by at least three orders.

The experimental setup that we have proposed only enables enlargement of X-ray diffraction patterns in one direction. Moreover, combining two identical setups does not make it possible to achieve enlargement in two perpendicular directions simultaneously, since it is impossible to simultaneously scan the slit and the X-ray film in two mutually perpendicular directions. However, in our earlier work (Drmeyan *et al.*, 2023 ▸), we proposed a scanning method that allows sequentially scanning the slit and the film, first in the horizontal and then in the vertical directions.

During the creation of the diffraction system under study, the required accuracy of thicknesses and equalities of interblock distances of the system crystalline blocks and their alignment (ideal geometry) were ensured, and due to mechanical collimation and the Bormann effect, the required collimation of the beams was ensured too. Therefore, the requirements for the accuracy of the system to conduct reliable experiments were fully fulfilled.

## Data Availability

Research data are available upon reasonable request to the first and corresponding authors.
